# Crayfish Self-Administer Amphetamine in a Spatially Contingent Task

**DOI:** 10.3389/fphys.2018.00433

**Published:** 2018-05-14

**Authors:** Udita Datta, Moira van Staaden, Robert Huber

**Affiliations:** Department of Biological Sciences, Bowling Green State University, Bowling Green, OH, United States

**Keywords:** addiction, amphetamine, invertebrate reward, crayfish, operant learning

## Abstract

Natural reward is an essential element of any organism’s ability to adapt to environmental variation. Its underlying circuits and mechanisms guide the learning process as they help associate an event, or cue, with the perception of an outcome’s value. More generally, natural reward serves as the fundamental generator of all motivated behavior. Addictive plant alkaloids are able to activate this circuitry in taxa ranging from planaria to humans. With modularly organized nervous systems and confirmed vulnerabilities to human drugs of abuse, crayfish have recently emerged as a compelling model for the study of the addiction cycle, including psychostimulant effects, sensitization, withdrawal, reinstatement, and drug reward in conditioned place preference paradigms. Here we extend this work with the demonstration of a spatially contingent, operant drug self-administration paradigm for amphetamine. When the animal enters a quadrant of the arena with a particular textured substrate, a computer-based control system delivers amphetamine through an indwelling fine-bore cannula. Resulting reward strength, dose-response, and the time course of operant conditioning were assessed. Individuals experiencing the drug contingent on their behavior, displayed enhanced rates of operant responses compared to that of their yoked (non-contingent) counterparts. Application of amphetamine near the supra-esophageal ganglion elicited stronger and more robust increases in operant responding than did systemic infusions. This work demonstrates automated implementation of a spatially contingent self-administration paradigm in crayfish, which provides a powerful tool to explore comparative perspectives in drug-sensitive reward, the mechanisms of learning underlying the addictive cycle, and phylogenetically conserved vulnerabilities to psychostimulant compounds.

## Introduction

The activation of natural reward pathways signifies the perception of a positive outcome in adaptive situations, such as when the individual manages to satisfy its demands for food, sex, or contact comfort (Kelley and Berridge, 2002). Prior studies have demonstrated that these circuits are sensitive to stimulation by a number of plant secondary compounds (Wink, 2015), even in the absence of any inherent beneficial outcomes (Koob, 2015). Cues experienced during such exposure, whether novel or previously encountered, acquire special salience and become labeled as rewarding. As vulnerable individuals enter an addictive cycle, they increasingly pursue conditions that enhance access to both the drugs themselves and the cues with which they are paired (Robinson and Berridge, 2000; Hyman and Malenka, 2001). The commonly accepted view that addiction is an exclusively human and cognitive phenomenon, is erroneous, and has for far too long hindered the emergence of a comprehensive understanding of addiction processes. The ability to duplicate drug-associated neural properties and behavioral consequences in other mammals, both primate and non-primate, resulted in the use of an expanded taxonomic range in preclinical addiction studies (Deneau et al., 1969; Collins et al., 1983; Bergman et al., 1989; Spealman et al., 1989; Sanchis-Segura and Spanagel, 2006). More recently, interest has focused on the potential utility of invertebrate model systems as we have come to appreciate that addiction represents a much more fundamental biological phenomenon of associative learning than had previously been thought. This perspective becomes somewhat less radical when one considers that the majority of addictive substances are defensive plant alkaloids to deter insect herbivory (Wink, 2015). Invertebrate models including *Drosophila*, honeybees, nematodes, and recent work on crayfish, have significantly enriched perspectives on addiction research. This ‘simpler systems’ approach (Wolf and Heberlein, 2003; Burne et al., 2011; Søvik and Barron, 2013; Yartsev, 2017) capitalizes on the structural efficiency, and unique accessibility to experimental manipulation that is inherent in invertebrate nervous systems. Most importantly, invertebrate and vertebrate models (humans included) are united by the conserved nature of reward mechanisms, sharing the same neurotransmitter systems with homo- and paralogous receptors, and featuring matched signaling pathways underlying behavioral addiction (Hen, 1992, 1993; Vernier et al., 1995, 1997; Porzgen et al., 2001; Tierney, 2001; Tierney et al., 2003).

A host of advantages make decapod crustaceans (i.e., crayfish, lobsters) a very suitable, and historically successful, model organism for exploring the neural machinery of behavior. Molecular, neurophysiological, and neurobehavioral experimentation (Clarac and Pearlstein, 2007) on the mechanisms of natural and drug-sensitive reward profits from a highly modular neural structure, conserved monoaminergic, neuromodulatory systems, a relatively small number of large and individually identifiable neurons, and high sensitivity toward human drugs of abuse. Amphetamine (Alcaro et al., 2011), cocaine (Nathaniel et al., 2012a,b), morphine (Nathaniel et al., 2010), and cathinones (Gore et al., unpublished data) exhibit potent psychostimulant properties, which sensitize with repeated exposure (Nathaniel et al., 2010, 2012b; Dziopa et al., 2011). Moreover, in a conditioned place preference paradigm (CPP), these substances trigger the formation of strong associations between drugs and the cues with which they are paired (Panksepp and Huber, 2004; Nathaniel et al., 2009). Discontinuing drug access produces withdrawal (Nathaniel et al., 2009; Huber et al., 2011), and a single, small priming dose is sufficient to fully reinstate a drug-induced CPP following a period of abstinence (Nathaniel et al., 2009). The present work expands on recent findings in which crayfish quickly learned to avoid areas paired with mild electric shock punishment (Bhimani and Huber, 2016). Here we advance a novel system for automated drug self-administration in crayfish, and explore whether, and to what extent, amphetamine reward alters crayfish behavior in an operant conditioning paradigm.

Conditioned place preference paradigm provides a measure for the reinforcing nature of a drug. However, because it relies on behavioral responses to conditioned stimuli, it is only an indirect assessment of a drugs affective properties. A more direct metric for an individual’s motivation to acquire drugs, and hence a drug’s inherent reward strength, derives from changes in operant behavior during a self-administration paradigm. In such a scenario the subject is able to control drug delivery by performing a learned, operant task (Gardner, 2000; Deroche-Gamonet et al., 2004; Belin et al., 2009), where successful task completion delivers a bolus of the substance. The ability to associate performance of the operant behavior and its earned drug infusion, is facilitated by both precise timing of drug delivery as well as by a rapid physiological response. Although a significant challenge for many smaller invertebrate study systems (Søvik and Barron, 2013), a rapid and precise drug delivery via an indwelling cannula is quite achievable in crayfish.

Using a fully-automated approach to crayfish behavior in a learned spatial task, we first assess baseline, unconditioned space use of an arena featuring distinct substrate textures. In a second step, we then reward each entrance into a particular substrate region with a bolus of drug. The study aims to determine whether individual crayfish can learn to perform tasks that gain them infusions of amphetamine by using their movement patterns to specifically revisit areas of the arena paired with drug. Effective demonstration of such an operant, self-administration paradigm would permit direct measurement and comparisons of relative reward strength of human drugs of abuse in crayfish, a quintessential model for behavioral neuroscience research.

## Materials and Methods

### Animals

Crayfish (*Orconectes rusticus*) were captured from the Portage River near Bowling Green, OH, United States (41.377965–83.475812). They were maintained in the laboratory under controlled environmental conditions in an aerated community tank (at 20°C, pH 7, 12 h L:12 h D) and fed twice a week with rabbit chow. Three days prior to the experiment, intermolt males (7–14 g) with all appendages intact were selected, individually housed in perforated plastic containers (Ø: 140 mm, ht: 70 mm), and placed in holding trays supplied with continuously circulating, filtered, aerated water from a large supply tank.

### Experimental Procedure

Training trials were performed in a circular polyethylene arena (Ø: 0.5 m, ht: 0.25 m) with the floor divided into four quadrants of two different substrates arranged diagonally. Tiles of white Plexiglas presented a smooth, hard surface, while tiles coated with a white, polyester mesh (Nonadhesive Easy Shelf Liner, Duck Brand, OH, United States) provided a soft, textured contrast. The arena was rotated between each trial. A custom, open-source video tracking solution (available for free download^1^) was used to record the movements of the test animal and to deliver a bolus of drug in real-time when the operant behavior was performed. Each experiment employed a new set of individuals, which were treated as described below.

Experiment 1 explored the unconditioned substrate preference, locomotion and arena use by drug naïve crayfish. For this experiment, spatial responses in two distinct groups of individuals that received saline injection either in the vicinity of supraesophageal ganglion (*n* = 6) or into the pericardium (*n* = 9) were recorded. Movements of saline treated individuals were recorded across a 5-h experimental time line. These provide the relevant baseline data for subsequent comparison with amphetamine-associated behavioral changes observed in Experiments 2 and 3.

The efficacy of amphetamine as a reinforcer under an operant conditioning paradigm was assessed in Experiments 2 and 3. In Experiment 2, the infusion cannula was implanted into the pericardial sinus for systemic application of drug at one of several dosages. Each experimental session lasted 3 h, during which movement of the individual into a quadrant with a particular texture earned a bolus of drug. Reinforcement was delivered for every instance of operant response. Following an operant response, a 5 s time timeout period was instated during which additional responses initiated did not result in drug infusions. Subjects (*n* = 12 per group) were randomly assigned to one of five drug dose categories. Under each dose category, animals were further classified either as: (1) Drug-Master individuals that received drug contingent to their entry into a particular substrate or (2) Drug-yoked animals that received an equal amount of amphetamine at the same time as the drug-master individual to which they were yoked. While the treatment animals had the opportunity to associate their action to the delivery of reward, individuals in the yoke group received drug infusions independent of their actions. Each drug dose level was thus evaluated in combinations of six master-yoke pairs. A saline group (*n* = 9) that received behaviorally contingent injections of saline served as the vehicle control.

The reward contingency for the two substrates (hard vs. soft) was counterbalanced among the individuals in each dose category. Learning of reward contingency was consequently evaluated for the hard substrate in three master- yoke pairs and for the soft substrate in another set of three master- yoke pairs. Experiment 3 was conducted in the same manner as that described above, except that the cannula for drug delivery was implanted directly over the supraesophageal ganglion (i.e., SEG, brain) of the crayfish. In this iteration the bolus was therefore delivered in close proximity to the neural tissues of the head ganglion, rather than reaching it indirectly via the general circulation. Previous work focusing on psychostimulant effects had demonstrated that injection in the head region resulted in stronger behavioral effects and a more rapid response for a given drug infusion (Alcaro et al., 2011).

### Surgery

**FIGURE 1:**
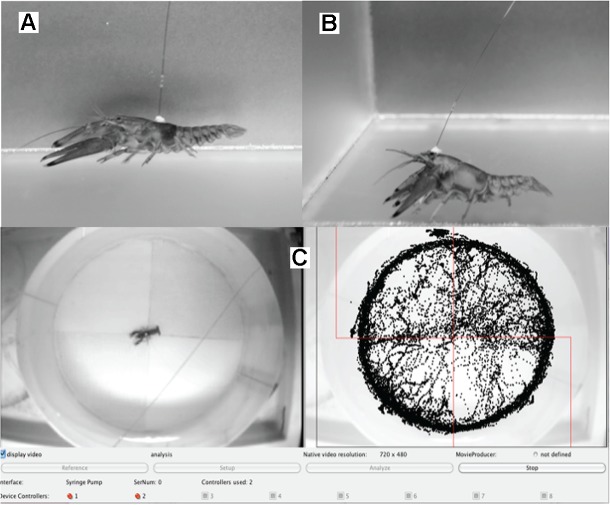
Positions of the cannula for the two different anatomical locations. Cannula implanted in the pericardial cavity **(A)** vs. in the vicinity of the supraesophageal ganglion **(B)**. Movements of the animal were monitored in real time and visualized using a computerized tracking framework. Drug reward was automatically delivered following the occurrence of an operant response. The quadrants with reward paired entries on the representative tracking window **(C)** are outlined in red. Location of the animal (depicted as black dots on the tracking window) captured at a sampling rate of 2 Hz. Time stamps, x and y Cartesian coordinates, and instances of operant responses were obtained and saved into a text file for subsequent analysis.

Prior to surgery, animals were cold anesthetized for 20 min in ice. Cannulae were implanted through the carapace to deliver drug either into the general circulation via the pericardial sinus (Experiment 2), or directly over the SEG (Experiment 3). Precise positioning of the cannula (**Figures 1A,B**) was informed through a series of preliminary dissections. For systemic infusion (Experiment 2) a 26.5 gauge needle was used to drill through the exoskeleton into the anterior end of the sinus, and slightly lateral of the midline, to avoid damaging the underlying heart. A 50 mm section of deactivated, fused silica material (Agilent 160-2655, i.d. = 50 μm, o.d. = 250 μm) was inserted through the opening such that 3 mm entered the pericardial sinus, and attached to the carapace with cyanoacrylate and bonding material. For Experiment 3 the cannula was placed over the SEG at the same insertion depth. Following surgery, the animals were allowed to recover overnight in their holding containers.

### Drug and Injection Protocol

Tygon microbore tubing (Fisher Scientific ND 100-80, i.d. = 250 μm) was used to connect a 0.5 m section of deactivated, fine-bore, fused silica needle material (Agilent 160-1010, i.d. = 100 μm, o.d. = 190 μm, 0.5 m long) to the implanted animal stub on one end and the blunt-tipped needle on a 1 ml glass syringe (SGE Analytical Sciences, Model# 008100) on the other. A syringe pump (Razel R-99E with R-ACC-6 Multi Micro Syringe Adapter) was positioned above the experimental arena, allowing concurrent drug application to multiple animals.

Doses of D-amphetamine sulfate (Sigma-Aldrich A 5880, St. Louis, MO, United States) were prepared in 125 mM saline (NaCl) and tested for their ability to support self-administration at two anatomical locations: pericardium (Experiment 2: five doses of amphetamine: 0.1, 0.3, 1, 3, and 10 μg/bolus), and supraesophageal ganglion (Experiment 3: three doses of amphetamine: 0.1, 0.3, and 1 μg/bolus).

### Behavioral Analysis

Movements of the animal within the experimental arena were captured using the JavaGrinders tracking framework. The analog signal from an overhead camera (Sony HDR-HC5 HDV 1080i) was digitized via an A/D converter (Canopus ADVC-110, 720x480 pixel resolution) on an Apple Macintosh computer (iMac, OSX 10.7.4). A collection of freeware programming functions for the analysis of behavior (available for free download^1^) were employed to capture time-stamped coordinates in a 2D Cartesian plane at a sampling rate of 2 Hz (**Figure 1C**). A minimum distance of 3.5 pixels between captures was required for inclusion as a movement event, to distinguish these from actions associated with grooming bouts. Operant tasks were defined as all instances in which the test individual crossed from an unpaired substrate into a reward paired one. The syringe was controlled by the tracking framework via a serial interface (USB/serial adapter DB-9RS-232). Each instance of operant response triggered the infusion of a 5 μl bolus containing a particular treatment delivered over a period of 1 s. This automated system offered reliable and rapid response-reward pairing over the course of extended trials. Movement descriptors, operant behaviors, and drug delivery were extracted post-trial from the time-stamped data logged to a file. Enhanced locomotion necessarily emerges from unconditioned psychostimulant effects and thus inevitably results in increased rates of operant responses. To distinguish between unconditioned and conditioned psychostimulant effects we calculated the number of valid responses per distance traveled as a measure of how effective movements were used to activate the pump [i.e., operant index (OI)].

### Statistical Analyses

Statistical analyses were conducted using R (Version 3.4.3, R Development Core Team, 2008). Levels of significance were set at *p* ≤ 0.05 for all tests. Substrate and quadrant preferences were assessed using a two-tailed, within subject design. Since OI values were neither normal (Shapiro–Wilk *W*-test, *p* < 0.001) nor homoscedastic (Brown–Forsythe test, *p* < 0.001), a conservative approach was adopted and original values of the variable were replaced by their rank equivalents. For Experiments 2 and 3, each 3 h experiment was binned into 20 min intervals and a mean OI was calculated for each time segment and effect of reward contingency tested with a repeated measures design.

## Results

### Experiment 1: Unconditioned Substrate Preference, Locomotion, and Arena Use

**FIGURE 2:**
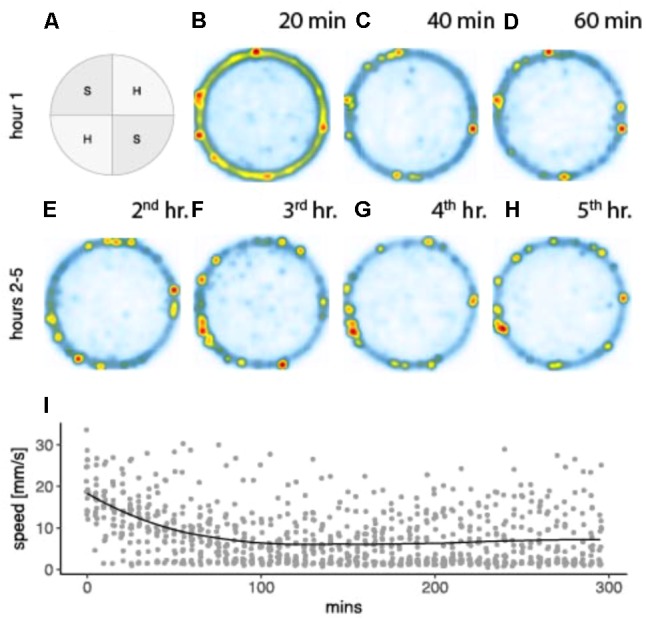
Spatial preferences combined for 15 drug naive crayfish in an arena with two soft and two hard textured quadrants arranged diagonally **(A)**. Heat maps depict utilization distributions obtained from two dimensional kernel density estimation (*kde2d*, package MASS, R Version 3.4.2) for 20 min segments of the first hour **(B–D)**, and 60 min segments for hours 2–5 **(E–H)**. Pixel densities range from low (white and blue) to high (yellow and red). Mean walking speeds for 5 min time segments are plotted for the 5 h time line with geometric loess smoothing and estimated standard error region **(I)**.

This study analyzed spatial responses in 15 saline treated individuals (six receiving brain infusions, nine receiving pericardial infusions) prior to, or in the absence of drug conditioning, across the 5-h experimental time line. These provide the relevant baseline data for subsequent analysis of amphetamine-associated behavioral changes, substrate preferences, locomotor activity, and space utilization summarized in **Figure 2**. When placed into the arena, drug-free crayfish spend much of their time following the circular outer wall, only occasionally leaving the periphery to cross the central, open portion of the arena. Initial walking speeds are consistent and high, occasionally interrupted by brief moments of hesitation when they approach the transition between substrate textures. Initial locomotion is paired with intense tactile and olfactory sampling indicative of exploration, but over the first hour mean speeds slow considerably as crayfish increasingly settle into stationary periods along the perimeter wall. Preferred places to settle appear to be the soft-textured side adjacent to a hard quadrant border. This is reflected in a significant preference for soft quadrants (mean p[soft] ± SE, *p* = 0.581 ± 0.015), which begins to emerge as a significant effect (one-sample *t*-test versus a hypothetical population mean *p* = 0.5, *t*_[14]_ = 5.2865, *p* < 0.001) 40 min into the trial. With their locomotor responses, control individuals earned saline infusions at a mean rate ( ± SE) of 30.43 infusions per hour. A repeated measures analysis confirmed that the rate of infusions was a direct linear function of locomotion (1.28 infusions per meter traveled *F*_[1,55]_ = 1840.834, *p* < <0001, adjacent *r*^2^ = 0.959) and that this relationship remained constant over the 5 h time period (*F*_[4,55]_ = 1.642, *p* = 0.177).

### Experiments 2 and 3: Unconditioned Psychostimulant Effects of Amphetamine

**FIGURE 3:**
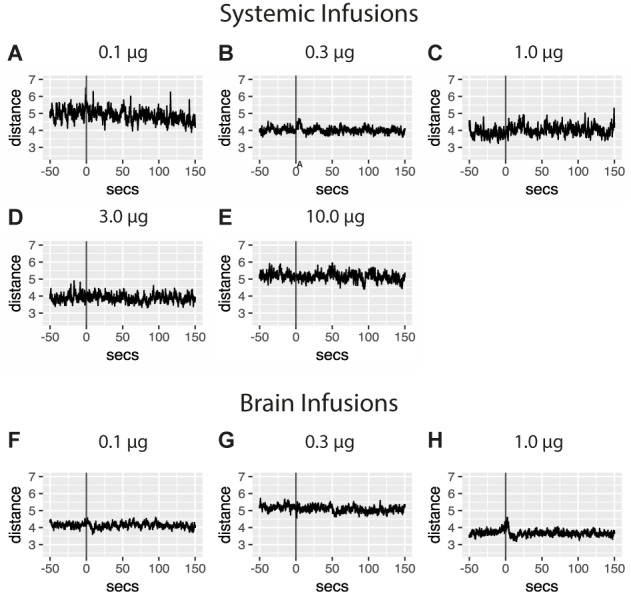
Unconditioned psychostimulant effects on locomotion were examined with stimulus averaging of repeated amphetamine infusions administered to yoked controls. Traces are aligned to the time of infusion (gray line) with average measures of locomotion plotted 50 s prior to and 150 s past the administration of a 5 μl bolus containing one of several drug amounts into the pericardial sinus **(A–E)** or brain **(F–H)**. No effects of pericardial infusions were detected although more subtle changes in traces may exist in **(A)** 0.1 μg (451 infusions in five individuals), **(B)** 0.3 μg (1321 infusions in six individuals), **(C)** 1 μg (521 infusions in five individuals), **(D)** 3 μg (566 infusions in five individuals), and **(E)** 10 μg doses of amphetamine (647 infusions in six individuals). Averaged traces for distance traveled following infusions above the crayfish brain of **(F)** 0.1 μg (1290 infusions in six individuals), **(G)** 0.3 μg (1042 infusions in six individuals), and **(H)** 1 μg amphetamine (927 infusions in six individuals). Traces indicate a brief, dose-dependent psychostimulant effect, followed by a short period of psychodepression.

Individuals in the yoked groups received amphetamine infusions contingent on their master’s operant responses and independent of their own behavior. In this group then, observed responses to the drug can thus inform amphetamine’s unconditioned behavioral effects. Individuals from the brain master group earned an hourly average of 43.1 (0.1 μg), 34.8 (0.3 μg), and 30.9 (1 μg/bolus) infusions respectively. Infusions at the highest dose are accompanied by a brief, dose-dependent psychostimulant effect, followed by a short period of psychodepression (**Figure 3**). Data also demonstrate that pericardial infusions of amphetamine were unassociated with distinct changes in levels of locomotion.

### Experiment 2: Pericardial Infusions – Effect of Reward Conditioning on Operant Responding

**FIGURE 4:**
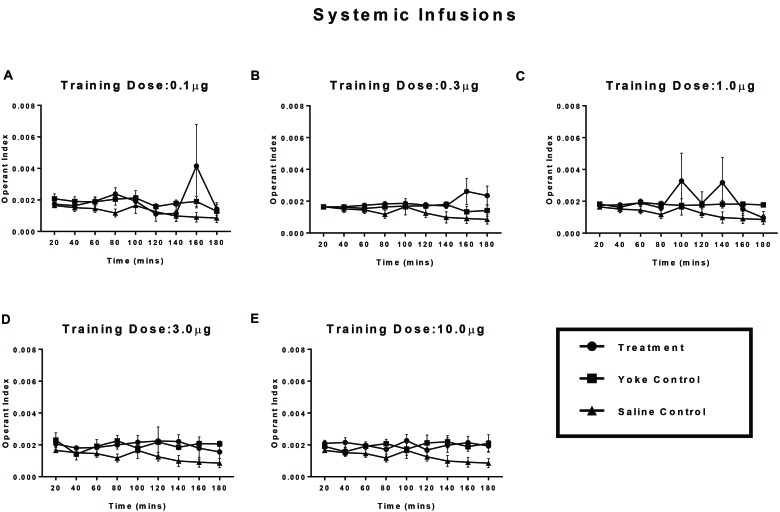
Operant conditioning of a spatial task paired with amphetamine reward, delivered into the pericardial sinus. Treatment animals received amphetamine infusions upon entering a chosen substrate. Yoked controls received the drug infusions at time points identical to their treatment partners. Vehicle controls received contingent saline infusions. Learning of reward contingency was compared using operant index (OI) between treatment and yoke groups for each dose category **(A–E)**. Operant conditioning in crayfish receiving behavior contingent injections of amphetamine at one of several doses **(A)** 0.1 μg, **(B)** 0.3 μg, **(C)** 1 μg, **(D)** 3 μg, and **(E)** 10 μg doses of amphetamine into the pericardium is less robust than animals receiving the drug over the supraesophageal ganglion. Mean OI for 20 min time segments are plotted for the 3 h session with standard error of mean.

Treatment and yoke pairs for each dose category (*n* = 6 pairs/dose) were compared based on OI (Experiment 2; **Figure 4**) using a within-subject design. No clear distinction in the levels of operant responding between treatment and their yokes was observed for any of the doses assayed. While higher OI scores of treatment relative to the yoked group were observed, most prominently at the drug dose of 0.3 and 1.0 μg/infusion, they failed to reach statistical significance. For comparable dose categories, systemic amphetamine injection produced less distinct differences in OI scores between self-administering and yoke groups relative to brain infusions.

### Experiment 3: SEG Infusions – Effect of Reward Conditioning on Operant Responding

**FIGURE 5:**
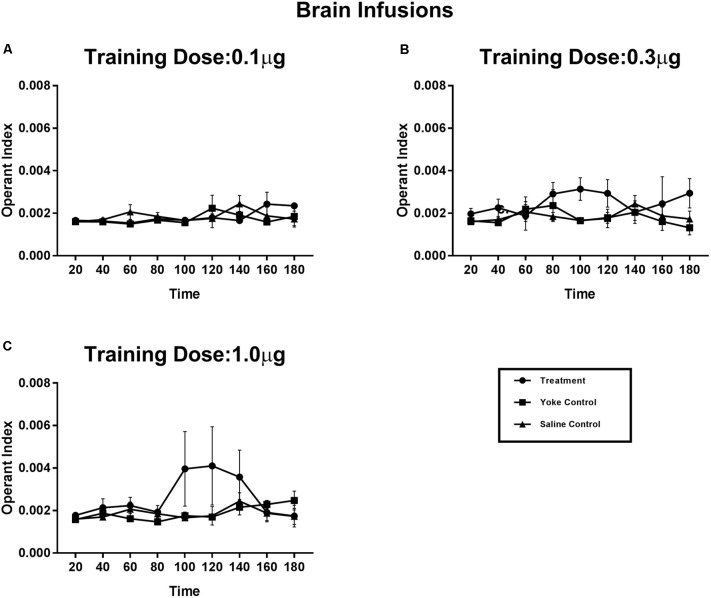
Operant conditioning of a spatial task using amphetamine reward delivered at the supraesophageal ganglion. Treatment animals received amphetamine infusions upon entering a chosen substrate. Yoked controls received the drug infusions at time points identical to their treatment partners. Vehicle controls received contingent saline infusions. Learning of reward contingency was compared using operant index (OI) between treatment and yoke groups for each dose category **(A–C)**. Increases in OI appeared in dose dependent manner. Lower doses of amphetamine **(A)** 0.1 μg, and **(B)** 0.3 μg per infusion display more subtle effects on operant index. Learning of reward contingency appeared prominently at the higher dose of **(C)** 1.0 μg per infusion, with self-administering animals selectively engaging in the drug paired behavior more than their yoked counterparts. Operant index scores of treatment group show an increase after 80 min of exposure to reward conditioning compared to the yoke group (Treatment X Time interaction: *F*[8,3] = 68.29, *p* < 0.05) indicating the time frame necessary for the learning of reward contingency. Mean Operant learning index for 20 min time segments are plotted for the 3 h session with standard error of mean.

The effect of injection site on reward strength was examined by comparing the previously described systemic injections (Experiment 2) with those infused near the brain (Experiment 3; **Figure 5**). Effects of reward contingency over the duration of the conditioning session was significant when examined using a repeated measures design (Treatment × Time interaction: *F*_[8,3]_ = 68.29, *p* < 0.05). OI scores of the treatment animals in the 1.0 μg dose group showed an increase after 1.5 h, whereas OI scores of the yoke remained unchanged across the trial. The evaluation of 3.0 and 10 μg/infusion doses were restricted to pericardial administration. When injected near the brain, these higher doses produced strong motor responses (including tail flips and excessive grooming), which precluded normal locomotion. The increase in OI scores of treatment- relative to the yoke groups was also observed for both the intermediate- (0.3 μg/infusion) and the lowest doses (0.1 μg/infusion) but was not statistically significant. OI scores of treatment and yoke groups appeared to be more similar when operant tasks were rewarded with lower doses of amphetamine, indicative of a dose-dependent increase in reward strength. The difference in OI scores between treatment and their yoked counterparts was maximum for the highest dose assayed.

## Discussion

Crayfish placed in a novel arena show enhanced levels of locomotion and antennal movements while actively exploring their surroundings. In the natural context, this active seeking drive is essential for encountering critical resources. As crayfish become familiar with their environment, a reduction in locomotion is observed, and animals tend to settle along the perimeter walls of the test arena. The ability of amphetamine to increase motor activity and stereotypy in mammals has been widely documented (Fog, 1969; Schiorring, 1971;Segal and Mandell, 1974; Hoebel et al., 1983). Using the distance traveled by yoke individuals that experienced the drug in a non-contingent fashion, we found that the unconditioned effects of the drug did not vary in a dose-dependent manner. Levels of locomotion were identical for all dose categories. A lack of amphetamine-induced increase in measures of locomotion for crayfish has previously been observed (Panksepp and Huber, 2004). One possible explanation for unchanged locomotory response level includes increased time spent in tactile exploration of the arena. In crayfish, exploration of surroundings is strongly dependent on mechanoreception using active movements of the antenna (Basil and Sandeman, 2000; Koch et al., 2006). Therefore, it is possible that stimulation of the appetitive motivational states by amphetamine results in increased tactile investigation of the surroundings via sensory appendages rather than increases in locomotion *per se*.

The present paper demonstrates the ability of crayfish to self-administer amphetamine in an operant conditioning paradigm. Free moving, behaving crayfish learn to self-inject amphetamine under continuous reinforcement schedules. We found the rewarding potential of amphetamine to be dose dependent, and the reward potency to vary with the site of injection. Injections near the supraesophageal ganglion exhibited stronger reinforcing qualities than did systemic infusions of the drug. With the establishment of a self-administration paradigm utilizing an automated and targeted drug delivery technique through implanted cannulae, we introduce an invertebrate system whose properties closely resemble those of mammalian self-administration models.

The ability of amphetamine to act as a reinforcer in the crayfish nervous system has previously been demonstrated using a CPP (Panksepp and Huber, 2004). In that study, amphetamine-evoked CPP appeared after just a single exposure, was persistent, and displayed prompt reinstatement. Here we have demonstrated that under a spatially contingent, operant conditioning paradigm, crayfish can learn to execute tasks paired with amphetamine infusions. Crayfish that experienced amphetamine reward contingent on their behavior displayed significantly higher OI scores. In contrast, yoked individuals that received amphetamine injections on the same temporal pattern but in a manner unrelated to their own behavior, did not display a similar increase. The vehicle control group that received contingent injections of saline also displayed no change in their OI scores across the session. Under the current paradigm, OI measures the individuals’ efficiency of movement to regulate self-administration through the activation of the infusion pump. The higher OI scores solely in individuals that were controlling their exposure to the drug (self-administering individuals) indicates that when crayfish are offered the opportunity to control delivery of drug reward, they will increasingly engage in behaviors that allows them to obtain the drug.

Operant conditioning using amphetamine reward in crayfish appeared in a dose dependent manner. Studies in rodent models have demonstrated that the rate and probability of acquisition of self-administration are positively correlated with the unit dose (van Ree et al., 1978; Carroll and Lac, 1997). Low unit doses of amphetamine (0.1 and 0.3 μg/infusion) were unable to act as a reinforcer of sufficient strength in our operant conditioning paradigm. Identical scores for OI were observed in self-administering individuals and their yoke at low unit doses, indicating that crayfish made no particular effort to self-administer the drug at these doses. Differences in OI scores were observed at the 0.3 μg unit dose but failed to achieve statistical significance. For 1.0 μg, the highest dose included in our study for supraesophageal ganglion drug administration, a significant increase in OI was observed in animals experiencing the reward contingently compared to their yokes. OI scorers in the self-administering group rose rapidly midway through the conditioning session, indicating learning of the reward contingency and the onset of active drug seeking.

Toward the end of the session, OI scores tended to decrease, suggesting that there is a ceiling for amphetamine intake which is likely a function of both the total amount of drug injected and the unit dose per injection. Plateauing amphetamine intake after a period of self-administration is indicative of a decrease in reinforcement efficacy, either because the amount of amphetamine injected established internal levels of the drug that reached satiation, or because they generated aversive states beyond a given level. Previous studies conducted in our lab have indicated that amphetamine at higher doses (5 mg/kg) increases the occurrence of tail flips and convulsions (Alcaro et al., 2011). Since tail flips are innate escape responses of crayfish employed under perceptions of serious threat, it is likely that at higher doses amphetamine generates aversive states that constrain further drug intake.

Self-administering individuals displayed higher variance in their OI scores compared to the yoke and vehicle control groups, as indicated by large error bars in the dose response curve. Large inter-individual differences in response to drugs have also been observed in humans and other animal models (de Wit et al., 1986; Piazza et al., 1998; Marinelli, 2005). Although self-administration may be acquired with relative ease by some individuals, others tend to be more resistant. Another factor potentially contributing to this large variance is the source of the sample. Since our *O. rusticus* sample is derived from a wild population, the error bars reflecting between-subject variability in the acquisition of operant responding are likely to be large.

Brain injections of amphetamine were self-administered more readily compared to systemic injections of amphetamine. Although systemic injections with a broad range of doses were tested, we observed few apparent changes in OI scores of the treatment groups relative to the yoke group. It was previously demonstrated that administration of D-amphetamine directly into the crayfish brain is more efficient than pericardial injections at enhancing exploratory behaviors (Alcaro et al., 2011). Cumulatively, these findings indicate that the potential target of amphetamine reward indeed resides in the crayfish brain. Application of the drug directly over the supraesophageal ganglion minimizes the time delay between operant response and the experience of reward thus increasing the effectiveness of the conditioning paradigm. Findings from both invertebrate (Kusayama and Watanabe, 2000; Panksepp and Huber, 2004; Carvelli et al., 2010; Alcaro et al., 2011) and vertebrate models highlight the role of the monoaminergic pathway in amphetamine reward (Sora et al., 2010; Howell and Negus, 2014; Wiers et al., 2016). In crayfish, monoamines have been demonstrated to modulate motor control, exploration, and more complex behaviors such as aggression and anxiety-like responses (Fossat et al., 2014). Considering the highly conserved functions of biogenic amines, they are also likely to play a role in reward processes in both natural and abnormal contexts (e.g., behaviors displayed under the influence of addictive drugs such as drug seeking, self-administration, and relapse). Both dopamine (Tierney et al., 2003) and serotonin (Sandeman and Sandeman, 1987; Sandeman et al., 1988) innervations occur prominently in the accessory lobe of crayfish. The accessory lobe, a structure capable of processing higher-order multimodal inputs, may thus be a critical brain region involved in the implementation of reward in crayfish.

Although the reward seeking circuit (Ferenczi et al., 2016; Otis et al., 2017) in crayfish brain is yet to be mapped out in its entirety. Nonetheless, with an amine system consisting of fewer than 1,000 neurons (30–35 dopamine neurons in the brain and nerve cord) and a well-characterized set of behaviors associated with drug reward, crayfish is a model amenable to the exploration of reward mechanisms (Shipley et al., 2017). The establishment of an automated, operantly conditioned self-administration paradigm in crayfish sets the stage for more nuanced studies of the processes underlying invertebrate reward. Such studies should aim to understand the implementation of an appetitive/seeking disposition in what is a relatively simple neural system, and by what particular mechanism/s this disposition is targeted by the rewarding action of drugs of abuse.

## Ethics Statement

Invertebrates are animals; however, no animal use protocols are required.

## Author Contributions

UD designed and carried out experiments, acquisition of data, analysis and interpretation of data, drafting and revising the article. RH helped with instrumentation and design, analysis, and interpretation of data. MvS assisted in conception and design, analysis and interpretation of data, drafting and revising of article.

## Conflict of Interest Statement

The authors declare that the research was conducted in the absence of any commercial or financial relationships that could be construed as a potential conflict of interest.

## Footnotes

1 http://iEthology.com/
